# Is Oxygen a Simple Body?

**Published:** 1895-11

**Authors:** 


IS OXYGEN A SIMPLE BODY?—Mr. E. C. C. Bally, preparator to Professor Ramsay, has just presented to the Royal Society of London a preliminary note tending to establish the fact that oxygen is not a simple body, as has hitherto been thought, but an association of two distinct gases. The fact that he announces is this: If oxygen be submitted to a silent electric discharge, the gas that goes to the cathode, according to the experiment, while remaining oxygen, exhibits a density sensibly different from that of non-electrified oxygen. In the case of long sparks, the density is less. The opposite is the case when short sparks are made to act. Is this as much as to say that the ordinary density of oxygen represents simply the major part of the density of the molecules of the gas, and that the silent discharge has the effect of sorting out such molecules in assembling those that are of the same weight?

